# Application of physics encoded neural networks to improve predictability of properties of complex multi-scale systems

**DOI:** 10.1038/s41598-024-65304-w

**Published:** 2024-07-01

**Authors:** Marcel B. J. Meinders, Jack Yang, Erik van der Linden

**Affiliations:** 1 Wageningen University and Research Centre, Wageningen, The Netherlands; 2 Wageningen Food and Biobased Research, Wageningen, The Netherlands; 3 Wageningen University, Wageningen, The Netherlands

**Keywords:** Neural networks, Machine learning, Physics encoded neural networks, Multi-scale modeling, Complex systems, Information theory and computation, Rheology

## Abstract

Predicting physical properties of complex multi-scale systems is a common challenge and demands analysis of various temporal and spatial scales. However, physics alone is often not sufficient due to lack of knowledge on certain details of the system. With sufficient data, however, machine learning techniques may aid. If data are yet relatively cumbersome to obtain, hybrid methods may come to the rescue. We focus in this report on using various types of neural networks (NN) including NN’s into which physics information is encoded (PeNN’s) and also studied effects of NN’s hyperparameters. We apply the networks to predict the viscosity of an emulsion as a function of shear rate. We show that using various network performance metrics as the mean squared error and the coefficient of determination ($R^2$) that the PeNN’s always perform better than the NN’s, as also confirmed by a Friedman test with a *p*-value smaller than 0.0002. The PeNN’s capture extrapolation and interpolation very well, contrary to the NN’s. In addition, we have found that the NN’s hyperparameters including network complexity and optimization methods do not have any effect on the above conclusions. We suggest that encoding NN’s with any disciplinary system based information yields promise to better predict properties of complex systems than NN’s alone, which will be in particular advantageous for small numbers of data. Such encoding would also be scalable, allowing different properties to be combined, without repetitive training of the NN’s.

## Introduction

Complex multi-scale systems are systems that are continuously changing, in an interdependent, self-organizing, and adaptive manner. An example of a real-life complex multi-scale system is the agri-food system^1^. A systematic way to predict the response of a complex multi-scale system on an intervention will involve analyses on its multiple scales, including the adaptive dynamics at all scales. This has been coined a complex systems approach^1^. Such approach has been argued to thrive from integrating various methodologies, including scaling, multi-scale modeling, machine learning, including graphical and evolutionary algorithms (^1^ and specific references therein). Also computational techniques have been suggested for the same application area by Perrot et al.^2^. However, in both articles, no specific methodology was articulated on how to specifically integrate multi-scale modeling and machine learning techniques in a quantitative fashion.

Conducting a multi-scale analysis is a complex endeavor since multiple connections can exist between different scales. Furthermore, such properties evolve, and the according dynamics usually cannot be covered by formal analytic descriptions. This according complexity makes the use of available mechanistic models alone difficult. A common strategy to relate scales with each other is to introduce intermediate length scales, which contain integrated information of the scales below, which can subsequently be incorporated into descriptions for the scale above the intermediate scale. An example is the so-called meso- or micro-structural scale in between the molecular and macroscopic scale. This strategy still has its challenges, attributed to the ill-reliability of microscale models, the difficulty in simulating the micro-scale properties accurately, and the often intricate “entanglement” between micro-structural and macroscopic scale properties^3^.

In order to accommodate these challenges described in the previous paragraph, machine learning (ML) using neural networks (NNs) can be an asset when sufficient data are available. In practice the amount of data available is not sufficient. In this case, using physical information may help. In general, combining physics-based multi-scale modeling with ML techniques may cleverly solve two problems at the same time. Physics may provide the structuring of ML techniques, and turning correlative relations into casual ones. Attempts to combine ML with physics-based modeling for dynamics of lake temperature^4–6^, and phosphorus concentration^7^ have shown that one can obtain better predictions with a smaller number of data and for scenarios that are distinct from the training scenario used in the ML algorithm itself. A recent review on physics-informed machine learning can be found in^8^ and with application focus on life sciences in^9^. In these reviews, the challenges and possible routes forward to model spatial-temporal evolution combining physics and ML are clearly addressed. To capture the spatio-temporal dynamics, one can use partial differential equations to express physics-based conservation laws, where such conservation laws can be constructed from constitutive laws that represent the local behaviour, in combination with using ordinary differential equations and their spatial derivatives. This has been illustrated in more detail for rheology-informed neural networks (RhiNNs) by Mahmoudabadbozchelou and Jamali^10^. Interestingly, Sadaat *et al*^11^ report the use of a platform of possible constitutive models to have the RhiNN pick from in order to optimize its predictions for more complex fluid behavior. These constitutive models need not represent the entire physics of the problem in all its details. Interestingly, parts of constitutive models can be captured by means of applying scattering under flow, as demonstrated by Young *et al* while using Small Angle X-ray scattering on dilute rod suspensions^12^. In trying to take into account spatio-temporal non-linear features, Dabiri *et al*^13^ report the use of fractional derivatives to incorporate into the NN models. Fractional derivatives are used to represent the presence of memory effects, which may be uncovered by introducing hidden variables that describe local effects, as addressed in Weinan *et al*^3^. In that work, an exciting example of introducing temporal (dynamical) information has been addressed in the form of so-called “recurrent neural networks”. The neural networks are machine learning models for time series. These models use hidden variables, making the relationships, as expressed in the models, local. If no hidden variables are being used, one effectively introduces memory effects^3^.

There are several ways at our disposal to add physical information into a neural network. For example, one can ascribe physics information to nodes in the network^14^. Alternatively, one can add physical information regarding symmetries that need to be obeyed. Another option could be the use of physics-based model data as input to AI models. For a survey on recent progress in various fields, the reader is referred to Willard *et al*^14^. It is noted that humans can develop physics-based architectures of neural networks, but this can be automated as well (^14^ references 13,73, 115). A concrete set of examples of improvement of neural network performance, which at the same time preserves the correctness of the physics, has been recently published by Takeishi and Kalousis^15^.

In regards to the agri-food area, in particular in applying ideas on combining multi-scale modeling with ML directly, i.e. without the need to reprogram the neural networks ML part, a review of Peng *et al*^16^ is worthwhile to mention. Works with more direct embedding in food science that address the combination of physics information and ML, without reprogramming the neural networks that underlie the ML are, for example, found elsewhere^17^. In this same area already some reviews can be found^18,19^.

We note that the above addresses adding disciplinary information to the input or output to an AI methodology. This is referred as physical informed neural networks (PiNNs) according to Faroughi *et al*^8^. On the other hand, physics knowledge can also be build into the network itself, referred to as physics encoded neural networks (PeNN’s)^8^. It is in this spirit that we like to situate our work on PeNN’s.

To our knowledge, encoding of physics information into NN’s in the area of multi-layer complex systems as described has not been addressed. Therefore, in the current article, we quantify the effects of including physics information in the architecture of NNs. We investigate uncertainty in prediction as a function of training set size, and effects on uncertainty/errors in inter- and extrapolating beyond a training set. We specifically look into the problem of protein stabilized oil droplets aggregating into clusters, and how the cluster size distribution in turn will determine the shear viscosity versus shear strain. The problem becomes complex as the flow influences the aggregation and vice versa and as one adds more experimental parameters that are known to influence the viscosity, such as the pH, protein type etc. The example lends itself to demonstrating the improvement of the predictability of a NN by integrating physics into it, i.e. transferring an NN to a PeNN.

## Flow of complex food fluids

Many complex foods are in fact dispersions, which are composed of fluid or solid particles dispersed in a fluid. Their flow behaviour under deformation is expressed by the viscosity. Texture, consumer acceptance, and processing conditions are strongly related to the viscosity. To optimize formulation, it is important to get insights in the dispersion’s viscosity and its controlling factors. Similarly, this also holds for non-food materials like e.g. paints, coatings, and cosmetics. Due to the complex nature of the dispersions, the viscosity depends on the rate of deformation. For our purpose, we focus here on shear deformation.

**Figure 1:**
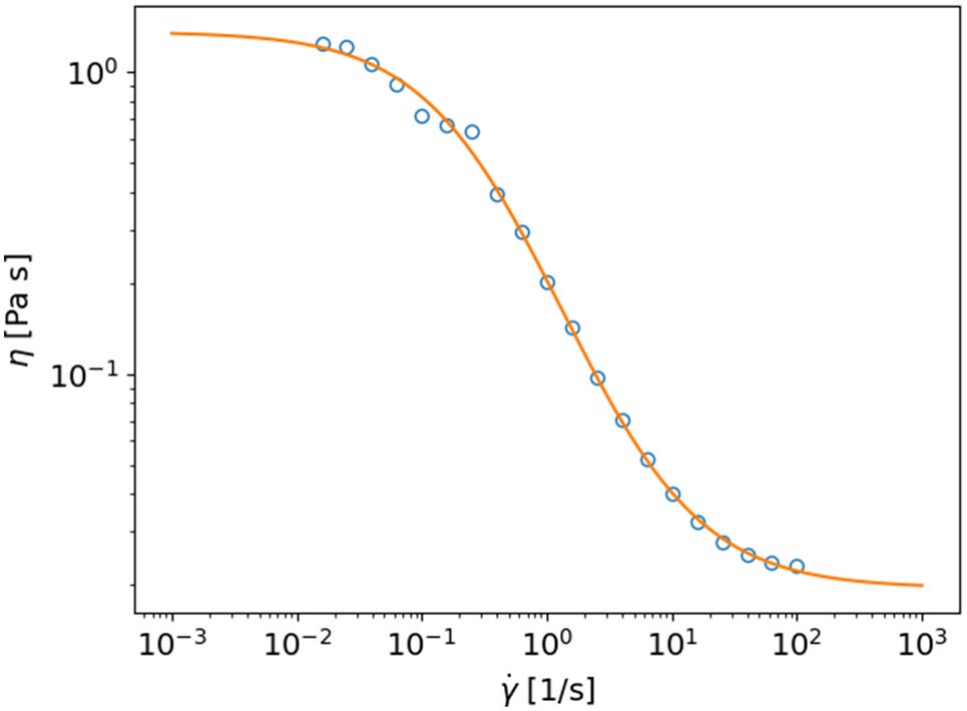
Example of a flow curve of an oil-in-water emulsion and Quemada-model-fit.

The shear rate-dependent viscosity depends on the structure of the dispersions and the interactions between the dispersed particles. The interactions control the assembly of (primary) particles into clusters. During deformation of the complex fluid, formation and breakdown of these clusters depend on time, shear rate, particle concentrations, cluster sizes, and inter-particle and inter-cluster interactions and temperature. Many studies have been published to describe the shear-rate dependent viscosity of complex dispersions^20–23^. Models to describe this kind of shear rate-dependent viscosity should contain the structural dynamics. One such model is the constitutive model by Quemada and coworkers^24–28^, which describes the rheology of complex colloidal systems in a large range of volume fractions using key physical parameters. Because the model fits very well with viscosities of food dispersions^20,21,23,29^ (see also Fig. 1), we used the Quemada model to study the predictive power of different NNs, and hybrid neural networks containing physics, for predicting the shear rate dependent viscosity of complex dispersions.

### Quemada model

The Quemada model describes the viscosity of complex fluids in terms of an effective volume fraction, which depends on the properties of the primary particles, including diffusion coefficients, interaction parameters, packing fraction of particles in a cluster, and shear rate. Here, we will focus on the semi-stationary regime, where the system is in equilibrium at a certain shear rate. The starting point of the model is the relation between the viscosity of the fluid $\eta$ and the effective volume fraction $\phi _e$. This relation is given by^24–28^

1 $$\begin{aligned} \eta = \eta _f \left( 1-\frac{\phi _e}{\phi _m} \right) ^{-2} \end{aligned}$$

with $\eta _f$ the viscosity of the continuous phase, and $\phi _m$ the maximum volume fraction. The effective volume fraction is given by^24–28^

2 $$\begin{aligned} \phi _e = \phi _{pA}/\varphi + (\phi _p-\phi _{pA}) = \phi _p \left( 1 + CS\right) \end{aligned}$$

with $\phi _p$ the volume fraction of the primary particles, $C=1/\varphi -1$, a compactness factor where $\varphi$ is the volume of the particles in a cluster divided by the volume of that cluster. *S* is a structural parameter defined as the ratio between the volume fraction of primary particles in the cluster $\phi _{pA}$ and the total volume fraction of primary particles $\phi _p$^24–28^

3 $$\begin{aligned} S = \frac{\phi _{pA}}{\phi _p} \end{aligned}$$

The structural parameter follows a certain kinetic reaction scheme, which, in its basic form, reads^24–28^

4 $$\begin{aligned} \frac{\textrm{d}S}{\textrm{d}t} = \kappa _D (S_0-S) - \kappa _h (S-S_\infty ) \end{aligned}$$

with $\kappa _D$ and $\kappa _h$ characteristic relaxation rates of Brownian (diffusion) and hydrodynamic (shear stress) forces. $S_0$ and $S_\infty$ correspond to the value of the structural parameter at zero and infinite shear rate. Additional terms related to particle interactions can be added^28^. The steady-state value of the structural parameter at a certain shear-rate $\dot{\gamma }$ is then given by^24–28^

5 $$\begin{aligned} S = \frac{S_0 + \theta S_\infty }{1+\theta } \end{aligned}$$

with

6 $$\begin{aligned} \theta = \frac{\kappa _h}{\kappa _D}=\frac{\dot{\gamma }}{a^2/D_p}=Pe=\frac{6\pi \eta _fa^3\dot{\gamma }}{k_BT} \end{aligned}$$

and *a* is the size of the primary particle, $D_p$ the diffusion coefficient of the primary particles, $k_B$ Boltzmann’s constant, *T* temperature, and *Pe* the Péclet number. The above equations give the fluid’s viscosity in the stationary state as a function of the key parameters^24–28^

7 $$\begin{aligned} \eta = \eta (\dot{\gamma }, \phi _p, S_0, S_\infty , C, \eta _f, a, T) \end{aligned}$$

Figure 1 shows an example of a measured flow curve of a pea protein stabilized oil-in-water emulsion, with an oil volume fraction of 0.5. The figure also shows the fit with the Quemada model with $\dot{\gamma }_c = \frac{6\pi \eta _fa^3}{k_BT}=4.7$ (corresponding to an average droplet size of $a=1$ $\mu$m), $CS_0=0.25$ (indicating that less than about 25% of the oil droplets are flocculated at zero shear rate) and $CS_\infty =0$ (indicating that all clusters broke up at a high shear rate).

### Neural networks and physics-encoded neural networks

To investigate the predictive power of NNs and PeNNs, shear rate dependent viscosity data was generated with the Quemada model and used to train and test NNs and PeNNs with different architectures. In order to align with physical experiments, we aim to predict $y=\eta$ from a set of input parameters $\{x_i\}$. In an experimental setting, $\eta$ is measured as a function of the shear rate $\dot{\gamma }$, so $\dot{\gamma }\in \{x_i\}$. Various other input characteristics of the dispersion can be measured or are known. For example, when preparing a sample, the volume fraction $\phi _p$ of the primary particles is known, like the volume fraction of oil in an emulsion. Other parameters can be measured and estimated using various different, often indirect, techniques, like e.g. the particle and cluster size distributions from light scattering techniques and inter-particle forces from DLVO theory.

The NNs and PeNNs were built using Python (version 3.9.16) in combination with the open source platform for machine learning TensorFlow (version 2.11.0), which uses Keras (version 2.11.0) as the high-level API. Data was generated and used to train various NNs and PeNNs with different architectures. The shear rate $\dot{\gamma }$ and viscosity $\eta$ can vary over several orders of magnitude. To train, validate, and test the NNs and PeNNs, we used $\log \dot{\gamma }$ and $\log \eta$, so that all input and output parameters are in the order of 1 to 10, and no normalization of the input and output data is needed. We performed some extra calculations using batch normalization within the neural network layers. However, it turned out that the performance of the networks did not improve.

Different architectures of NN’s were investigated, varying in number of hidden layers and number of neurons per layer. The rectified linear (ReLu) activation function was used in all layers except for the final layer, for which we used the linear activation function because we are dealing here with a regression problem^30–32^ and not a classification problem. As default we used the Adam optimization algorithm to train the NN’s, which is generally accepted to be one of the most efficient algorithms in machine learning. We used a learning rate of $10^{-3}$ with a decay of $5\cdot 10^{-6}$, mean squared error (mse) as loss function or performance metric, and 20% of the learning data for validation. The training was stopped when the loss function of the validation set has stopped improving in 500 epochs using the build-in TensorFlow callback function Early-Stopping.

We also performed hyper parameter tuning by investigating different NN’s with different complexities, optimization routines like Nadam, AdamW, and Lion, learning rates, and activation functions.

We investigated two cases to compare. In case one, we predict the viscosity as a function of two input parameters, while in case two, we predict the viscosity as a function of four input parameters.

Performance of the NNs and PeNNs was assessed using the performance metrics coefficient of determination $R^2$ and the mean square error (mse) between predicted and ground truth viscosity values of the test set, for unbiased evaluation. To check to what extent the performance of the studied networks differ, a graphical comparison of the metrics was performed. We also includied a Friedman test to quantify the performance differences, similar as done by Zamri *et al*^33^. In addition, we compared the predicted and ground truth viscosities graphically.

#### Case 1: networks with 2 input parameters and 1 output parameter

**Figure 2:**
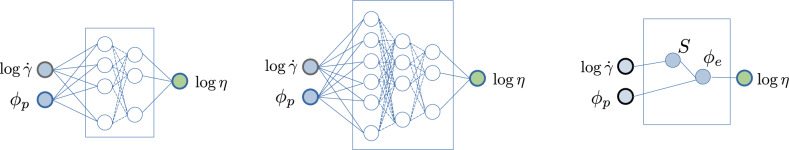
Schematic architecture of the NN (left, middle) and PeNN (right) configuration.

For case one, two different NNs were studied (see Fig. 2). One with an architecture consisting of two hidden dense layers. The first layer has $n_1=32$ neurons and is densely connected to the $n_{in}=2$ input nodes, while the second layer has $n_2=8$ neurons. Another NN studied has an architecture consisting of three hidden dense layers. The first, second and third layer has $n_1=128$, $n_2=32$ and $n_3=8$ neurons, respectively. For both NNs, the first layer is densely connected to the $n_{in}=2$ input nodes, while the last layer is densely connected with the output layer having $n_{out}=1$ neuron.

The PeNN consists of 3 layers, each corresponding to a physical quantity, in other words, the activation functions are completely physics-based. In this sense, this PeNN is actually totally dominated by physics, to illustrate the importance of physics in a NN. The first layer corresponds to the structure parameter *S*. It has one input $x=\log \dot{\gamma }$ and activation function $S_{act}= 1/(10^xw + 1)$. Here, *w* is a trainable parameter and should correspond to $\frac{6\pi \eta _fa^3}{k_BT}$ as can be simply derived from Eqs. 5 and 6. The second layer corresponds to the effective volume fraction $\phi_e$ having two inputs, being the output of the S-layer and the input $\phi_p$. The activation of the $\phi_e$-layer is $\phi _{e,act}= x_1(x_2w + 1)$, with input $\varvec{x} = [x_1, x_2]$ and *w* a trainable parameter corresponding to *C* (equation 2). The third layer corresponds to the viscosity with activation function $\eta_{act} = -2\log (1-xw)+b$, where *w* and *b* are trainable parameters, corresponding to $1/\phi_m$ and $\log \eta _f$, respectively, and *x* is the input equal to the output of the effective-volume-fraction-layer.

In case one, only the shear rate $\dot{\gamma }$ and particle volume fraction $\phi _p$ were varied and taken as input parameters for the constitutive model to generate the steady-state fluid viscosity $\eta$. Other parameters were taken constant, being $T=293$ K, $a=5$ nm, $C=2$, $S_0=1$, $S_\infty =0$, and $\eta _f=10^{-3}$ Pa.s.

#### Case 2: networks with 4 input parameters and 1 output parameter

**Figure 3:**
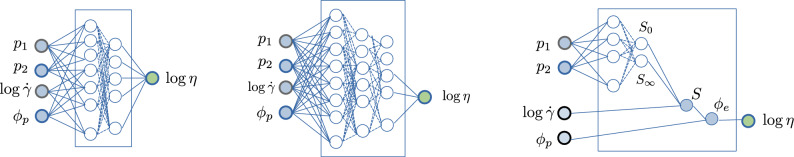
Schematic architecture of the NN (left, middle) and PeNN (right) configuration.

In case 2, also $S_0$ and $S_\infty$ were varied to generate flow curve data to train NNs and PeNNs with architectures, as depicted in Fig. 3. These parameters correspond to the structure parameter at zero and infinite shear rate, respectively. In this sense, this PeNN is not totally dominated by physics. In general, parameters are difficult to asses as they can be related to handling history, inter-particle forces, amount of protein denaturation, pH, salt concentration, etc. Here, we took two representative input parameters $p_1$ and $p_2$ between 0 and 1.

Similar as above, two different NNs were studied with architectures consisting of two and three dense hidden layers consisting of 32-8 and 128-32-8 neurons. For both, the first layer is densely connected to the $n_{in}=4$ input nodes, while the last layer is densely connected with the output layer having $n_{out}=1$ neuron.

The PeNN consist of 6 layers, of which the first 3 are a dense connected NN with outputs that should mimic the structure factor at low and high shear rate $S_0$ and $S_\infty$, respectively. The following and last 3 layers correspond each to the physical quantities, as explained above. The only exception is that the S-layer now has three inputs $\varvec{x} = [x_1 x_2 x_3]$ corresponding to $[S_0 S_\infty \log \dot{\gamma }$] and physics-based activation function $S_{act}= (x_1 +10^{x_3}wx_2)/(10^{x_3}w + 1)$. Here, again *w* is a trainable parameter and should correspond to $\frac{6\pi \eta _fa^3}{k_BT}$. The last layers are the same as described above.

#### Data sets

For both cases 1 and 2, various data sets were generated and used to train and test the performance of the NNs and PeNNs, varying in number of input parameters (as discussed above) as well as varying in number of data points per input parameter. The generated data was split into a training set (75%, randomly chosen) used to train NNs and PeNNs, and a test-holdout set (25%) to test the performance after training. During training, 20% of the set was used for validation.

It is noted that in rheology experiments, in general the number of data points for the shear rate $\dot{\gamma }$ is much larger than that for a parameter like the volume fraction of the primary particles $\phi _p$. This is because it is rather simple to obtain 100 or more data points ($\eta (\dot{\gamma })$) in a viscosity measurement for just one sample (with a certain $\phi _p$). For better comparison with real life experiments we therefore generated the data sets in a similar way: for each of *n* different $\phi _p$’s (with $\phi _p \in \{\phi _1 \ldots \phi _n\}$), *N* different $\log \dot{\gamma }$ (with $\dot{\gamma }\in \{\dot{\gamma }_1 \ldots \dot{\gamma }_N\}$ were chosen as input parameters, with $N>>n$. This yields $n\times N$ data triplets ($\eta (\phi _p,\dot{\gamma })$ for case 1, and $n\times N \times n_{p1} \times n_{p2}$ data quintets ($\eta (\phi _p,\dot{\gamma }, p_1, p_2)$ for case 2. Here $n_{p1}$ and $n_{p2}$ are the number of input parameters $p_1$ and $p_2$ respectively. Here we choose $n_{p1}=n_{p2}=2$. The generated data set was randomly split into a training set (75%, thus 0.75*nN* and $0.75nNn_{p1}n_{p2}$ data points for case 1 and 2, respectively) and a test-holdout set (0.25*nN* and $0.25nNn_{p1}n_{p2}$ data points for case 1 and 2, respectively). In general, the test-holdout set is used to check the performance of a NN to unseen data. Although the NN did not see the data triplets ($\eta (\phi _p,\dot{\gamma })$ of the test set, it did see numerous data with $\phi _p\in \{\phi _1 \ldots \phi _n\}$. In order to check to what extend the NNs and PeNNs can also generalize to unseen volume fractions, thus how they perform for $\phi _p \notin \{\phi _1 \ldots \phi _n\}$, another set, referred to as the test set, was created by choosing randomly $n_{tst}=10^4$ input parameters $(\phi _p,\log \dot{\gamma })$ with $\phi _p$ between $\phi _{p,min}=0.01$ and $\phi _{p,max}=0.2$ and $\log \dot{\gamma }$ between $\log \dot{\gamma }_{min}=-5$ and $\log \dot{\gamma }_{max}=3$. This test set ($10^4$ data points for case 1 and 2) was used for an unbiased comparison of the performance of the networks.

The pseudo code of the algorithms used can be found in the appendix.

## Results

### Case 1: networks with 2 input parameters and 1 output parameter

**Figure 4:**
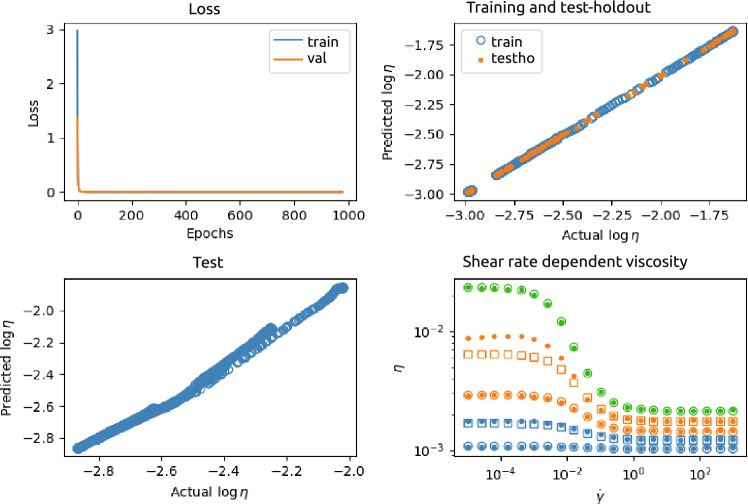
Results of the NN 2-128-32-8-1 neural network (see Fig. 3). This NN was trained and tested using a data set generated from $n=3$ different $\phi _p$ and for each $\phi _p$ $N=300$ different $\dot{\gamma }$. Top-left: loss as a function of number of epochs; Top-right: predicted versus actual values of the training (blue) and test-holdout (orange) set; Bottom-left: predicted versus ground truth of the test set containing unseen $\phi _p$; Bottom-right: examples of ground truth (actual) shear rate dependent viscosity ($\circ$ (seen $\phi _p$) and $\square$ (unseen $\phi _p$)) and predicted shear rate dependent viscosity ($\bullet$). The colors indicate different $\phi _p$.

Figure 4 shows an example of the results of a NN for case 1, with two input nodes ($\phi _p$ and $\log \dot{\gamma }$), three hidden layers with 128, 32, and 8 neurons, respectively, and one output layer $\log \eta$. This NN was trained and tested using a data set generated from $n=3$ different $\phi _p$ and for each $\phi _p$ $N=300$ different $\dot{\gamma }$. The top-left panel shows the loss function as a function of the number of epochs. The top-right panel shows the predicted output as a function of the actual (ground truth) output, for the training set (675 data points) as well as the test-holdout set (225 data points). The predicted values and actual values of the validation set ($10^4$ data points), also containing $\phi _p$-values not seen by the NN, are plotted against each other in the left-bottom panel. In addition, the right-bottom panel of the figure shows the predicted and ground truth viscosity as function of shear rate and for different $\phi _p$, seen, as well as unseen during training.

**Figure 5:**
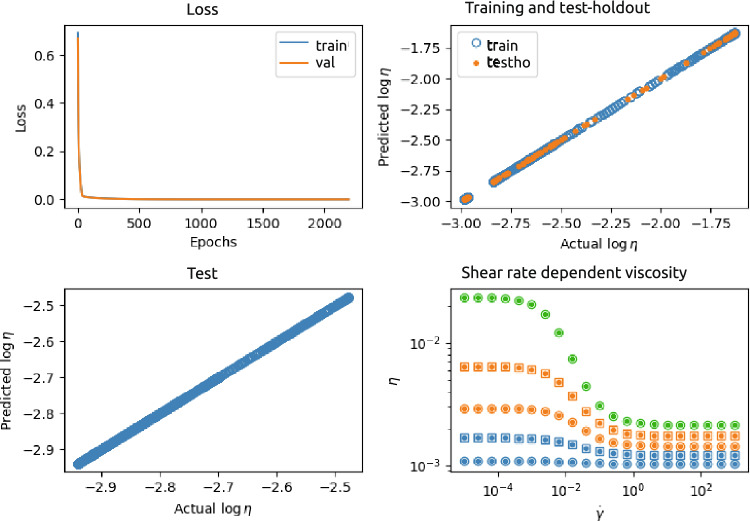
Results of the PeNN 2-1-1-1 [physical-encoded neural network (see Fig. 3). This NN was trained and tested using a data set generated from $n=3$ different $\phi _p$ and for each $\phi _p$ $N=300$ different $\dot{\gamma }$. Top-left: loss as a function of number of epochs; Top-right predicted versus actual values of the training (blue) and test-holdout (orange) set; Bottom-left: predicted versus actual values of the test set containing unseen $\phi _p$; Bottom-right: examples of actual shear rate dependent viscosity ($\circ$ (seen $\phi _p$) and $\square$ (unseen $\phi _p$)) and predicted shear rate dependent viscosity ($\bullet$). The colors indicate different $\phi _p$.

Figure 5 shows an example of the results of the PeNN for case 1, with two input nodes ($\phi _p$ and $\log \dot{\gamma }$), two layers completely physics-encoded, and one output layer $\log \eta$. The top-left panel shows the loss function as a function of the number of epochs. The top-right panel shows the predicted output as a function of the actual (ground truth) output, for the training set (675 datapoints) as well as the test-holdout set (225 data points). The predicted values and actual values of the test set ($10^4$ data points) containing $\phi _p$’s not seen by the PeNN are plotted against each other in the left-bottom panel. In addition, the right-bottom panel of the figure shows the predicted and ground truth (actual) viscosity as function of shear rate and for different $\phi _p$, seen, as well as unseen during training.

We conclude that in this case 1, replacing neural nodes by physics does improve NNs significantly. The viscosity as function of shear rate and primary particle volume fraction are predicted very well, also for values of input parameters which the model has not been trained for. Thus interpolation and extrapolation are captured well for the PeNNs.

### Case 2: networks with 4 input parameters and 1 output parameter

**Figure 6:**
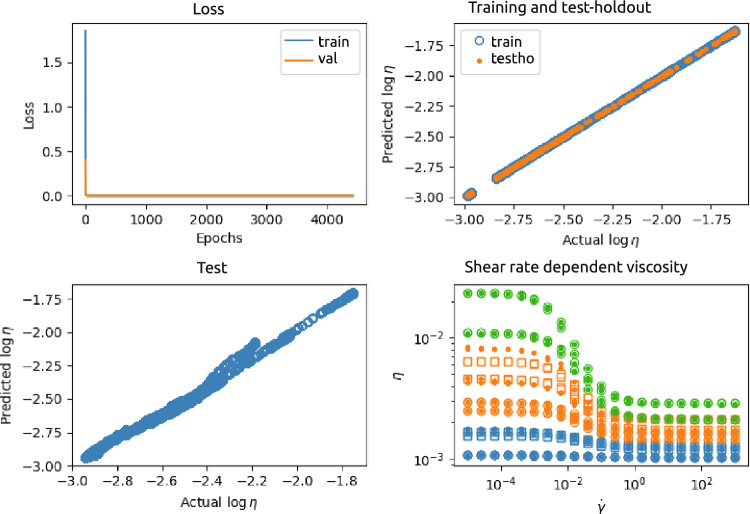
Results of the NN 4-32-8-1 neural network (see Fig. 3). The NN was trained and tested using a data set generated from $n=3$ different $\phi _p$ and for each $\phi _p$ $N=300$ different $\dot{\gamma }$. Top-left: loss as a function of number of epochs; Top-right predicted versus actual values of the training and test-holdout set; Bottom-left: predicted versus actual values of the test set; Bottom-right: examples of actual shear rate dependent viscosity ($\circ$ (seen $\phi _p$) and $\square$ (unseen $\phi _p$)) and predicted shear rate dependent viscosity ($\bullet$). The colors indicate different $\phi _p$.

Figure 6 shows an example of the results of a NN for case 2, with four input nodes ($\phi _p$, $\log \dot{\gamma }$, $p_1$, and $p_2$), two hidden layers with 32 and 8 neurons, respectively, and one output layer $\log \eta$. This NN was trained and tested using a data set generated from $n=3$ different $\phi _p$ and for each $\phi _p$ $N=300$ different $\dot{\gamma }$ and $n_{p1}=n_{p2}=2$ different values for $p_1$ and $p_2$ respectively. The top-left panel shows the loss function as a function of the number of epochs. The top-right panel shows the predicted output as a function of the actual (ground truth) output, for the training set (2700 data points) as well as the test-holdout set (900 data points). The predicted values and actual values of the test set ($10^4$ data points) are plotted against each other in the left-bottom panel. In addition, predicted and actual curves with different $\phi _p$, seen, as well as unseen during training, as function of $\dot{\gamma }$ are shown in the right-bottom panel.

**Figure 7:**
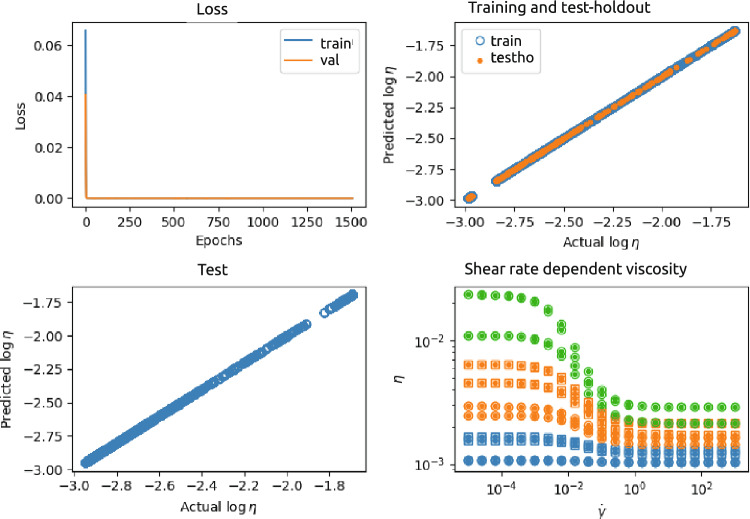
Results of the PeNN 4-6-2-1-1-1 physical-encoded neural network (see Fig. 3). The PeNN was trained and tested using a data set generated from $n=3$ different $\phi _p$ and for each $\phi _p$ $N=300$ different $\dot{\gamma }$. Top-left: loss as a function of number of epochs; Top-right: predicted versus actual values of the training (orange) and test-holdout (blue) set; Bottom-left: predicted versus actual values of the test set $\phi _p$; Bottom-right: examples of actual ($\circ$) and predicted ($\bullet$) flow curves (colors indicate $\phi _p$.

Figure 7 shows an example of the results of the PeNN for case 2, with four input nodes ($\phi _p$, $\log \dot{\gamma }$, $p_1$, and $p_2$), 2 dense NN-layers and 3 layers completely physics-encoded, of which the last one is the output layer $\log \eta$. The top-left panel shows the loss function as a function of the number of epochs. The top-right panel shows the predicted output as a function of the actual (ground truth) output, for the training (2700 data points) and test-holdout set (900 data points), while the left-bottom panel shows this for the test set ($10^4$ data points). The right-bottom panel of the figure shows the predicted and ground truth viscosity as function of shear rate and for different $\phi _p$, seen, as well as unseen during training.

We conclude also for case 2 that replacing neural nodes with physics does improve the NNs significantly. The viscosity as function of shear rate and primary particle volume fraction are predicted very well, also for values of input parameters which the model has not been trained for. Thus interpolation and extrapolation are captured well for the PeNNs.

**Figure 8:**
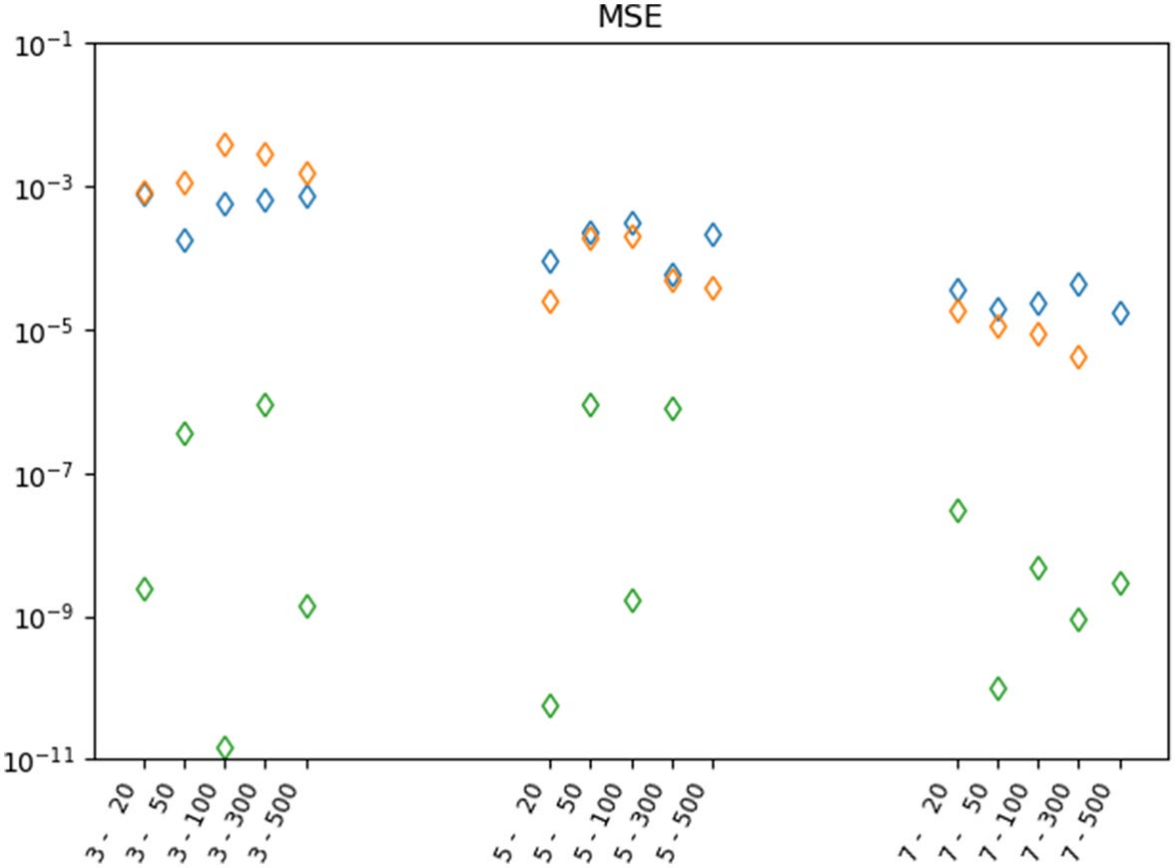
Mean squared error (mse) of the different NN’s and PeNN between predicted and ground truth values for the test set. The colors correspond to NN 4-32-8-1 (blue), NN 4-128-32-8-1 (orange), and PeNN 4-6-2-1-1-1 (green). The horizontal axis correspond to different data sets generated with $n\in [3,5,7]$ different $\phi _p$’s and for each $\phi _p$, $N\in [20, 50, 100, 300, 500]$ different $\log \dot{\gamma }$.

The observation that PeNNs perform better than NNs is also nicely illustrated in Fig. 8, showing the mean squared error (mse) for the NNs and PeNN for the test set. The figure shows that the mse of the PeNN is always at least about a factor 100 to 1000 smaller than that of the NNs. This indicates that the PeNN's performes significantly better than the NN’s. This is also confirmed by the Friedman test performed on the data displayed in Fig. 8. The Friedman test resulted in rejection of the H0 hypotheses (there is no performance differences between the models) with a p-value smaller than 0.0002. A Wilconox signed-rank test shows that the performances of all NNs are equal (not significantly different, with p-values larger than 0.6) while the PeNN performs significantly better than the NNs, with according p-values smaller than 0.002. We refer to Supplementary Information for more details.

We also studied the effect of NN’s hyperparameters (different architectures with different complexities, optimization algorithms, learning rates) as well as different performance metrics and have found that these have no effect on our main conclusion about the differences between performance of NN’s and PeNN's that PeNN’s outperform NN’s. We refer to Supplementary Information for more details.

## Discussion, conclusions, and perspective

We have illustrated for a complex multi-scale problem how physics information can be encoded into a neural network and how that leads to improvement of the predictability and robustness of the network. Similar results have been obtained in another field giving better predictions and with a smaller number of data^4–7^.

Our work uses physics to improve a NN model. The incorporation of physics into the architecture of the NN is such that the resulting architecture of the NN has the same architecture of the physics-based hierarchical architecture. It is this architectural hierarchical feature that is important in realizing the so-called Physics-encoded NN (PeNN). The term physics-encoded has some specific implications, which are clearly different from the features of other types of hybrid NNs, as also discussed by Faroughi *et al*^8^. Advantages of PeNNs above other forms of neural networks that are enriched with physics information are the efficiency of algorithms in finite dimension settings, robustness against data scarcity, and their modular transferability into other areas. Similarly, neural-mechanistic hybrid approaches have been utilized in genome-scale metabolic models recently by Faure *et al*^34^.

The physics-encoded NNs allows continued learning as opposed to instance learning. Instance learning implies that it is required to retrain an entire network for predicting outcomes in a new setting for that network. Continued learning resembles a feature of intelligence. In this view, physics-encoded neural networks should not be viewed as an “AI” model but an “I” model, i.e. leaving out the term “Artificial”. In respect to learning, it is noteworthy that recently Zador^35^ has argued that one may distinguish several levels of learning within animals, as opposed to the technical usage of the term learning within NN’s. Within animals, there exist learned and innate mechanisms in executing functions. The innate mechanisms are encoded in the genome, which provides the rules that wire up the brain, for the behavioral programs for many functions (walking, swimming, etc.). The wiring of the brain is not explicitly programmed but will evolve during development, on the basis of a set of rules given by the coding. Interestingly, artificial NNs have to be optimized according to what is learned during their evolution and the learning during their functioning. In contrast, animals learn only in their functioning, as the innate part has been encoded. In analogy to the above, in physics-encoded NNs, the physics provides the set of rules, that allow for the explicit efficient wiring of the NNs during their training period. In this sense, one always should use NNs that build on previous solutions (use their learning). This represents a “real” learning phase where information is stored in a structured manner, suitable for building new information on top of that information, in a congruent manner, instead of building a structure all the time on the basis of data available, en re-iterating this latter learning. In fact, the physical information encoding implies a form of “real” intelligence. This explains the advantage of using physics to encode the NN wiring. This wiring is not random but instead is containing information, obtained from exposure to its surroundings and subsequently storing that information, which, if used again, explicates learning. This view is extended into a formal theory of clever computing of systems as expressed by Jaeger *et al*^36^, where the ideal computing of systems occurs as a bottom-up activity that structures the processes along which the computing takes place (cybernetic), using physically observables (physics-encoded), instead of the classic computing systems that describe the processing along structures that are present (algorithmic).

Our work is straightforward and uses essential physics to improve a NN model. The NNs can predict the viscosity curves very well, when the input parameters are part of the training set. Predictions are significantly worse for parameters not in the training set. Thus a NN does not sufficiently capture interpolation and extrapolation. The physics model may not be entirely covering all aspects, but the physics will be directing the number of possibilities for the NN while optimizing during its learning stage. The incorporation of the physics into the architecture of the NN such that the resulting architecture of the NN has the same architecture of the physics-based hierarchical architecture. It is this architectural characteristic that is important in being embedded into the NN, which is different from what has been reported until now.

Using physics-based information introduces information in NN’s that has an experimental basis. The information added incorporates the existing and known natural sequence of events and their hierarchy, i.e. it incorporates the way that nature shows itself to us. In the same spirit as encoding physics information into NN’s (PeNN’s), one can encode information from other disciplines into NN’s, creating in general “Information encoded NN’s, or IeNN’s. Subsequently, one can imagine building different IeNN’s on top of one another, addressing even more complex systems.

### Supplementary Information

Supplementary Information.

## Data Availability

The datasets used and/or analysed during the current study is available from the corresponding author on reasonable request.

## Appendix

The pseudo code of the program can be found in Table 1

**Table 1:** Pseudo code of the program used

**Main algorithm**	
**Define** data [*n*], [*N*], $\dot{\gamma }_{min}$, $\dot{\gamma }_{max}$, $\phi _{pmin}$, $\phi _{pmax}$, $S_0$, $S_\infty$, *C*, $\eta _f$, *a*, *T*, $p_1$ and $p_2$	
[NNarchitectures, PeNNarchitecture], [hyper parameters]	
**Loop** over [*n*] and [*N*]	
Generate list of particle volume fractions $[\phi _p]$ of length *n* with $\phi _{pmin}< \phi _p<\phi _{pmax}$	
Generate list of shear rates logarithms $[\log (\dot{\gamma })]$ of length *N* with $\dot{\gamma }_{min}< \dot{\gamma }<\dot{\gamma }_{max}$	
Generate list of Quemada viscosities logarithms $[\log \eta ([\dot{\gamma }], [\phi _p], S_0, S_\infty , C, \eta _f, a, T)]$ of length *nN*	
Construct input data tensor *X* from $[\phi _p]$ and $[\log (\dot{\gamma })]$ with shape equals (*nN*, 4)	
Construct output data tensor *Y* from $[\log \eta ]$ with shape equals (*nN*, 1)	
Split *X* and *Y* randomly in training set $X_{train}$ and $Y_{train}$ (80%)	
and test-holdout set $X_{test-holdout}$ and $Y_{test-holdout}$	
Generate list $[\phi _p]_{test}$ of randomly chosen $\phi _{pmin}< \phi _p<\phi _{pmax}$	
Generate list $[\log \eta ([\dot{\gamma }]_{test}, [\phi _p]_{test}, S_0, S_\infty , C, \eta _f, a, T)]_{test}$	
Construct input data test tensor $X_{test}$ from $[\phi _p]_{test}$ and $[\log (\dot{\gamma })]_{test}$	
Construct output data test tensor $Y_{test}$ from $[\log \eta ]_{test}$	
Generate list $[\phi _p]_{t2}=[\phi _p]+[\phi _p]_{2}$ with list $[\phi _p]_{2}$ containing $\phi _p$’s not in $[\phi _p]$	
Generate list $[\log \eta ([\dot{\gamma }], [\phi _p]_{t2}, S_0, S_\infty , C, \eta _f, a, T)]_{t2}$	
Construct input data test2 tensor $X_{t2}$ from $[\phi _p]_{t2st}$ and $[\log (\dot{\gamma })]$	
Construct output data test2 tensor $Y_{t2}$ from $[\log \eta ]_{t2}$	
**Loop** over network models *list* [NNarchitectures, PeNNarchitecture]	
Build model	
Compile model with using hyper parameters for loss function and optimization algorithm	
Train (Fit) network model using training set and hyper parameters for validation	
and early-stopping callback giving trained model $\mathcal {M}$	
Calculate predicted outcome of training set $\hat{Y}_{train} = \mathcal{M}(Y_{train})$	
Calculate predicted outcome of test-holdout set $\hat{Y}_{test-holdout} = \mathcal{M}(Y_{test-holdout})$	
Calculate predicted outcome of test set $\hat{Y}_{test} = \mathcal{M}(Y_{test})$	
Calculate predicted outcome of test2 set $\hat{Y}_{t2} = \mathcal{M}(Y_{t2})$	
Calculate performance metrics $mse_\mathcal{M}$ and $R^2_\mathcal{M}$ from $\hat{Y}_{test}$ and $Y_{test}$	
Plot loss function as function of epochs	
Plot $\hat{Y}_{train}$ vs. $Y_{train}$ and $\hat{Y}_{test-holdout}$ vs $Y_{test-holdout}$	
Plot $\hat{Y}_{test}$ vs. $Y_{test}$	
Plot $\hat{Y}_{t2}$ vs. $Y_{t2}$	
Plot performance metrics for all models and all test sets like $mse_{\mathcal{M}, n, N}$	
Perform Friedman and Wilconox test	

The parameters and symbols are also used and explained in the manuscript. With [ ] a list is indicated.

## Code availability

The code used during the current study is available from the corresponding author on reasonable request.
