# Supplementary material for: Application of physics encoded neural networks to improve predictability of properties of complex multi-scale systems
Source: Sci Rep. 2024 Jul 1;14:15015. doi: 10.1038/s41598-024-65304-w (PMC11217277; doi:10.1038/s41598-024-65304-w)
Supplement: Supplementary file 1 — Supplementary Information. [file 41598_2024_65304_MOESM1_ESM.pdf]

Supplementary Information
Using Physics Encoded Neural Networks to improve predictability of properties of a complex multi-scale system.

Marcel B.J. Meinders[1,2,*], Jack Yang[1,3], and Erik van der Linden[1,3]

1 Hyper parameter investigation

1.1 Effect of NN complexity

Different architectures of NN's were investigated, varying in number of hidden layers and number of neurons per layer. Supplementary figure 1 shows the mean square error (mse) and the coefficient of determination R^2 of the NNs studied, applied to the test set, as a function of the complexity of the NNs. The mse is defined as

$$\text{mse} = 1/m \sum_i^m (y_i - Y_i)^2 \tag{1}$$

with Y_i and y_i the ground truth and predicted values, respectively, and m the number of data points in the test set. The coefficient of determination R^2 is defined via

$$1 - R^2 = \frac{\sum_i^m (y_i - Y_i)^2}{\sum_i^m (y_i - \langle y_i \rangle)^2} \tag{2}$$

with $\langle y_i \rangle$ the average of y_i

The complexity is defined as the number of neurons in the hidden layers. To train, validate and test the NNs data sets as described in the manuscript with $n = 3$ and $N = 300$. The NN's investigated in supplementary figure 1 are dense NNs, all with 4 input layers and 1 output layer and number of hidden layers varying between 1 and 6 and number of neurons per hidden layer between 2 and 128.

The rectified linear (ReLu) activation function was used in all layers except for the final layer, for which we used the linear activation function. Furthermore, the Adam optimization algorithm was used to train the NN's with a learning rate of 10^{-3} with decay and decay of $5\dot{1}0^{-6}$ The training was stopped when the loss function of the validation set has stopped improving in 500 epochs.

To compare better the difference, $1 - R^2$ is plotted instead of R^2.

1.1.1 Result and conclusion

It is seen that the values of these performance metrics are for all studied NNs larger than about 10^{-4}, while for the PeNNs these are below 10^{-4}. It can therefore concluded that the complexity of the NN's does not affect our main conclusion: PeNNs outperform NNs.

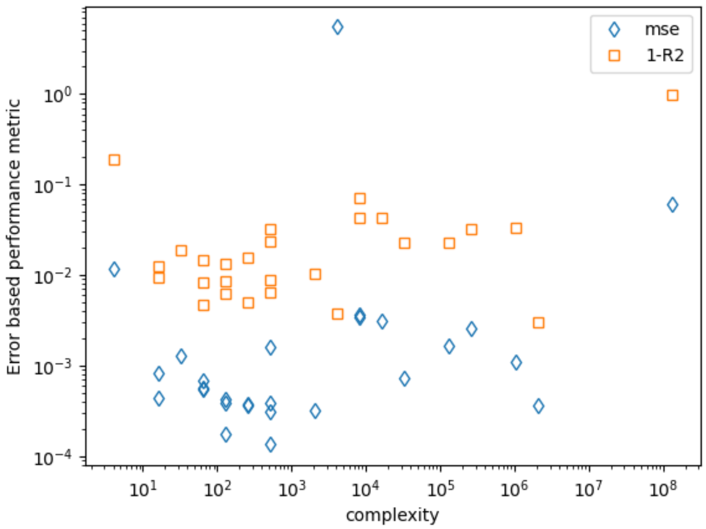

Supplementary figure 1: Performance metrics mse (blue diamond) and $1 - R^2$ (orange square) between predicted and ground truth values for the test set of various NN's differing in complexity (number of neurons in the hidden layers).

1.2 Effect of optimization method

We also studied the effect of optimization routines on the performance of the NN's. Therefore a NN with 4 input layer, 3 hidden layers with 128, 32, and 8 neurons, respectively, and 1 output layer are trained, validated and tested using data sets as described in the manuscript with $n = 3$ and $N = 300$. The training was performed using different optimization algorithms, being Nadam, AdamW, and Lion. The default settings of TensorFlow are used. Results are shown in supplementary figure 2

1.2.1 Result and conclusion

As above, it is seen that the values of these performance metrics are for all studied optimization routines are than above about 10^{-4}, while for the PeNNs these are below 10^{-4}. It can therefore concluded that the optimization routine does not affect our main conclusion: PeNNs outperform NNs

1.3 Effect of learning rates and activation functions

Also small studies are performed (results not shown here) with varying activation functions of the first layer and different learning rates and its decay using in the Adam optimization algorithm. For all these studies the mse of the test set was always larger than 10^{-4}

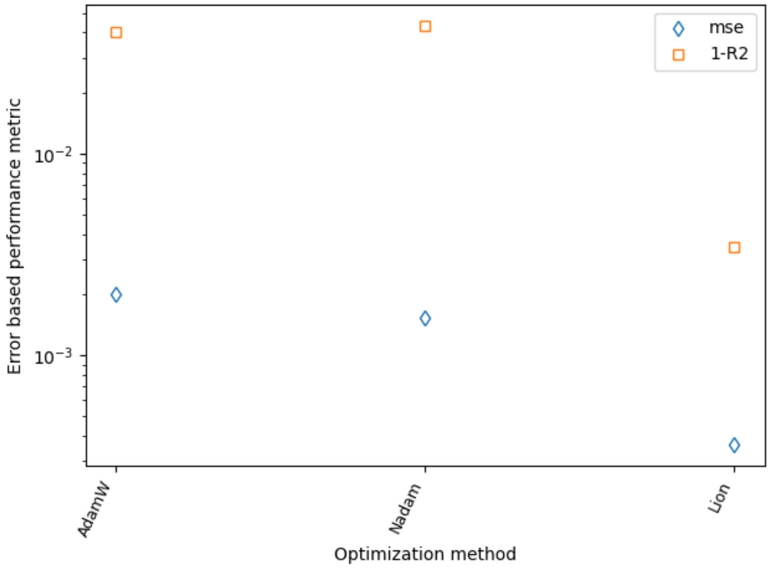

Supplementary figure 2: Performance metrics mse (blue diamond) and $1 - R^2$ (orange square) between predicted and ground truth values for the test set of NN's with 4 input layer, 3 hidden layers with 128, 32, and 8 neurons, respectively, and 1 output layer (NN 4-128-32-8-1). The NN's are trained and validated using different optimizations algorithms, as displayed on the horizontal axis.

2 Conclusion

We found that difference in NN architectures, NN complexities, optimization algorithms, learning rates, the use of different performance metrics do not have an effect on the main conclusion of the paper, which states that the performance of PeNN's are significantly better than that of NN's.
